# Effect of alirocumab on specific lipoprotein non-high-density lipoprotein cholesterol and subfractions as measured by the vertical auto profile method: analysis of 3 randomized trials versus placebo

**DOI:** 10.1186/s12944-016-0197-4

**Published:** 2016-02-13

**Authors:** Peter P. Toth, Sara C. Hamon, Steven R. Jones, Seth S. Martin, Parag H. Joshi, Krishnaji R. Kulkarni, Poulabi Banerjee, Corinne Hanotin, Eli M. Roth, James M. McKenney

**Affiliations:** CGH Medical Center, 101 East Miller Rd, Sterling, Illinois 61081 USA; University of Illinois School of Medicine, Peoria, IL USA; Johns Hopkins University School of Medicine, Baltimore, MD USA; Regeneron, Tarrytown, NY USA; University of Texas Southwestern Medical Center, Dallas, TX USA; Atherotech, Birmingham, AL USA; Sanofi, Paris, France; The Sterling Research Group, Cincinnati, OH USA; Virginia Commonwealth University, Richmond, VA USA

**Keywords:** Alirocumab, Hypercholesterolaemia, Lipoprotein subfraction, PCSK9, VAP

## Abstract

**Background:**

The effect of alirocumab on potentially atherogenic lipoprotein subfractions was assessed in a *post ho*c analysis using the vertical auto profile (VAP) method.

**Methods:**

Patients from three Phase II studies with low-density lipoprotein cholesterol (LDL-C) ≥2.59 mmol/L (100 mg/dL) at baseline on stable statin therapy were randomised to receive subcutaneous alirocumab 50–150 mg every 2 weeks (Q2W) or 150–300 mg every 4 weeks (according to study) or placebo for 8–12 weeks. Samples from patients treated with alirocumab 150 mg Q2W (*n* = 74; dose common to all three trials) or placebo (*n* = 71) were analysed by VAP. Percent change in lipoprotein subfractions with alirocumab vs. placebo was analysed at Weeks 6, 8 or 12 using analysis of covariance.

**Results:**

Alirocumab significantly reduced LDL-C and the cholesterol content of subfractions LDL_1_, LDL_2_ and LDL_3+4_. Significant reductions were also observed in triglycerides, apolipoproteins CII and CIII and the cholesterol content of very low-density, intermediate-density, and remnant lipoproteins.

**Conclusion:**

Alirocumab achieved reductions across a spectrum of atherogenic lipoproteins in patients receiving background statin therapy.

**Trial registration:**

Clinicaltrials.gov identifiers: NCT01288443, NCT01288469, NCT01266876

**Electronic supplementary material:**

The online version of this article (doi:10.1186/s12944-016-0197-4) contains supplementary material, which is available to authorized users.

## Background

Lipoproteins consist of lipids and apolipoproteins and can be categorised by density into five major classes: chylomicrons, very low-density lipoprotein (VLDL), low-density lipoprotein (LDL), intermediate-density lipoprotein (IDL) and high-density lipoprotein (HDL) [1]. Elevated levels of LDL cholesterol (LDL-C) are a well-established risk factor for the development of cardiovascular disease [2]. LDL-C is composed of a spectrum of LDL particles of different densities and states of lipidation, all of which are atherogenic [3]. Levels of the most dense LDL-C particles may be disproportionately raised in patients with low HDL cholesterol (HDL-C) and high triglycerides [4]. Analysis of changes in LDL subfractions may provide additional information to help direct individual patient treatment [5], although it is currently not recommended in guidelines [6].

In addition to LDL-C, triglyceride-rich remnant lipoproteins (RLP) are also atherogenic [4]. RLPs are products of VLDL lipolysis, and include VLDL_3_ (the smallest and most dense particles of the VLDL subclass) and IDLs (the direct precursor to LDL formation). Serum concentration of RLPs are often increased in patients with elevated triglyceride levels, such as those with lipoprotein lipase deficiency, insulin resistance, and metabolic syndrome. Two important regulators of triglyceride levels are apolipoprotein (apo) CII and CIII. ApoCIII inhibits lipoprotein lipase-mediated catabolism of VLDL triglycerides [7]. ApoCII appears to have a more complex relationship with VLDL and lipoprotein lipase activity that may depend on baseline triglyceride levels, and is generally an important activator of lipoprotein lipase [8].

Ultracentrifugation is the standard for direct measurement of LDL-C levels, as Friedewald calculation frequently underestimates LDL-C at levels <100 mg/dL or in the setting of elevated triglycerides [9]. Vertical auto profiling (VAP), a rapid and highly sensitive direct ultracentrifugation method, provides measurements of lipoproteins and subfractions not assessed using standard techniques [10–12].

Proprotein convertase subtilisin/kexin type 9 (PCSK9) regulates levels of the LDL receptor [13]. Alirocumab is a fully human monoclonal antibody to PCSK9 which significantly reduced LDL-C levels in clinical trials, with overall adverse events comparable to control [14–19]. In addition, significant reductions in apoB, non-HDL-C and lipoprotein(a), trends for reduction in triglycerides, and modest increases in HDL-C and apoAI, were observed.

PSCK9 exerts complex regulatory functions on lipoprotein metabolism [13]. PCSK9 inhibition correlates with substantial reduction in serum LDL-C levels [14–19]. We hypothesized that these large reductions in LDL-C would coincide with reductions in serum levels of all apoB-containing lipoproteins including remnant lipoproteins, possibly because of increased clearance of these lipoproteins by increased availability of LDL receptors.

Using VAP testing, a *post hoc* analysis of three alirocumab Phase II trials was performed to determine if the significant reductions in LDL-C observed with alirocumab were reflected by reductions across the spectrum of atherogenic LDL subfractions, and to determine the impact on other lipoproteins such as IDL cholesterol (IDL-C) and VLDL cholesterol (VLDL-C).

## Results

In the three Phase II trials, 108 patients received alirocumab 150 mg Q2W and 77 received placebo. Baseline characteristics from these patients are shown in Table 1. Baseline and post-treatment samples were available for VAP analysis from 100 alirocumab patients and 71 placebo patients.

**Table 1:** Baseline characteristics

	Placebo (*n* = 77)	Alirocumab 150 mg Q2W (*n* = 108)
Age, mean (SD), years	53.8 (9.4)	58.2 (10.1)
Males, %	49.4	43.5
Race, %		
White	84.4	88.9
Black or African American	13.0	11.1
Other	2.6	0
Body mass index, mean (SD), kg/m^2^	28.9 (4.7)	29.2 (4.6)
Lipid parameters (determined by conventional methods), mean (SD), mg/dL		
LDL-C	130.6 (27.5)	127.2 (25.0)
Total cholesterol	210.5 (32.1)	208.0 (30.5)
HDL-C	51.8 (13.8)	53.8 (15.0)
Non-HDL-C	158.7 (30.8)	154.2 (31.3)
Triglycerides, median (Q1:Q3)	122.5 (91.5–174.0)	123.5 (87.8–168.5)
Apolipoprotein B	108.8 (22.8)	108.1 (23.9)
Lipoprotein (a), median (Q1:Q3)	19.0 (6.0–77.0)	28.0 (9.0–70.0)

Pooled patients from placebo and alirocumab 150 mg Q2W groups of three Phase II studies. To convert cholesterol values to mmol/L, multiply by 0.02586; to convert triglycerides to mmol/L, multiply by 0.01129

*Q2W* every 2 weeks, *HDL-C* high-density lipoprotein cholesterol, *LDL-C* low-density lipoprotein cholesterol, *SD* standard deviation

### LDL-C and subfractions

Significant reductions in the cholesterol content of LDL and LDL_1–3_-C were observed in patients receiving alirocumab vs. placebo (Table 2). Baseline levels of the densest LDL subfraction (LDL_4_-C) were relatively very low. Percentage changes in levels of LDL_4_-C were inconsistent between studies, with high standard deviation values, although absolute reductions in LDL_4_-C were significant vs. placebo for all alirocumab groups (Table 2). When LDL_4_-C was assessed in a pooled analysis with LDL_3_-C (as LDL_3+4_-C; the two densest subfractions), mean reductions of 68.5, 55.2 and 48.8 % were observed in studies 565, 566 and 1003, respectively (*p* < 0.0001 vs. placebo). Reductions in LDL-C and subfractions were relatively large in the placebo group in study 566 (where the atorvastatin dose was increased at randomisation) compared with the other studies (in which patients received stable background statin therapy) (Table 2). Results were comparable between study 1003 (conducted in heFH patients) and studies 565 and 566 (conducted in non-FH patients) (Table 2). LDL-C reductions estimated using the Friedewald equation in the parent studies were 72.4 % (study 565), 66.2–73.2 % (study 566) and 67.9 % (study 1003; Additional file 1: Table S1). Reductions of 67.5, 62.2–64.6 and 60.3 % were observed in directly measured LDL-C in studies 565, 566 and 1003, respectively, and reductions of 74.9, 68.7–71.6 and 64.7 %, respectively, were observed in LDLr-C (Table 2).

**Table 2:** Changes from baseline in cholesterol content of LDL subfractions

	Study 565		Study 566^a^			Study 1003	
	Placebo	Alirocumab 150 mg Q2W	Placebo + ATV 80 mg	Alirocumab 150 mg Q2W + ATV 10 mg	Alirocumab 150 mg Q2W + ATV 80 mg	Placebo	Alirocumab 150 mg Q2W
	*n* = 31	*n* = 29	*n* = 26	*n* = 26	*n* = 29	*n* = 14	*n* = 16
Total LDL-C							
Baseline	115.2 (27.3)	109.6 (24.8)	120.1 (24.4)	123.0 (24.0)	122.1 (33.4)	141.4 (34.1)	135.7 (28.2)
Post-treatment^b^	111.1 (25.1)	34.5 (14.9)	97.7 (37.7)	43.5 (15.3)	44.6 (25.5)	130.4 (32.0)	53.4 (34.2)
% change from baseline	−1.1 (21.3)	−67.5 (13.6)**	−17.3 (29.9)	−64.6 (11.9)**	−62.2 (22.5)**	−6.9 (13.7)	−60.3 (25.8)**
LDLr-C							
Baseline	90.5 (25.0)	85.6 (22.5)	95.0 (22.3)	96.9 (21.7)	96.0 (29.2)	110.3 (28.9)	103.6 (24.1)
Post-treatment^b^	87.2 (21.6)	21.5 (12.5)	75.9 (32.4)	27.7 (12.6)	29.6 (22.5)	100.6 (24.4)	36.8 (29.9)
% change from baseline	0.1 (24.6)	−74.9 (14.4)**	−18.6 (32.5)	−71.6 (11.5)**	−68.7 (22.9)**	−7.1 (15.5)	−64.7 (28.6)**
LDL_1_-C							
Baseline	18.7 (6.3)	16.2 (5.6)	19.8 (7.0)	19.2 (7.7)	19.7 (9.2)	23.3 (10.2)	25.9 (9.9)
Post-treatment^b^	17.0 (6.9)	3.6 (2.8)	14.7 (9.2)	5.3 (3.5)	4.9 (4.9)	21.9 (10.8)	6.8 (6.2)
% change from baseline	−7.5 (24.4)	−76.9 (20.8)**	−18.4 (59.4)	−72.5 (16.4)	−40.7 (201.0)	1.0 (39.1)	−68.9 (41.3)**
LDL_2_-C							
Baseline	22.3 (11.7)	20.3 (10.9)	29.4 (13.8)	24.5 (12.3)	27.6 (15.6)	30.7 (17.5)	31.5 (17.1)
Post-treatment^b^	17.8 (12.5)	3.0 (4.4)	21.5 (16.1)	4.5 (4.9)	5.5 (8.4)	27.5 (13.7)	7.8 (11.9)
% change from baseline	2.9 (140.9)	−84.2 (26.2)*	−24.7 (48.5)	−83.7 (13.0)**	−83.4 (20.0)**	−2.6 (39.1)	−77.9 (26.1)**
LDL_3_-C							
Baseline	39.1 (15.2)	37.1 (13.5)	37.6 (14.8)	40.6 (14.0)	37.2 (16.4)	43.7 (13.2)	36.6 (12.5)
Post-treatment^b^	38.2 (12.2)	9.1 (6.6)	30.5 (13.2)	11.0 (6.3)	11.7 (10.1)	39.9 (12.0)	15.2 (12.8)
% change from baseline	4.7 (31.6)	−74.8 (20.4)**	−5.5 (63.5)	−71.5 (18.0)**	−64.9 (29.7)**	−5.0 (25.9)	−58.1 (33.9)**
LDL_4_-C							
Baseline	10.4 (7.1)	12.1 (9.0)	8.3 (6.4)	12.7 (8.5)	11.6 (11.6)	12.7 (9.7)	9.7 (11.4)
Post-treatment^b^	14.2 (9.2)	5.8 (2.5)	9.2 (5.0)	7.0 (3.1)	7.5 (4.0)	11.2 (6.7)	7.1 (3.5)
Absolute change from baseline	3.9 (8.5)	−6.2 (8.4)**	0.9 (4.7)	−5.7 (6.5)*	−4.1 (9.3)*	−1.4 (7.5)	−2.6 (9.7)*
% change from baseline	84.7 (164.0)	−28.6 (56.4)*	106.2 (236.9)	−5.6 (133.5)	51.3 (352.1)	12.8 (67.4)	53.7 (156.8)
LDL_1+2_-C							
Baseline	40.9 (16.6)	36.5 (13.9)	49.1 (17.5)	43.6 (17.9)	47.2 (21.5)	54.0 (25.2)	57.4 (24.9)
Post-treatment^b^	34.8 (17.8)	6.6 (6.2)	36.2 (24.2)	9.8 (7.9)	10.4 (12.0)	49.4 (21.5)	14.7 (17.6)
% change from baseline	−10.3 (41.2)	−81.0 (18.9)**	−22.6 (51.5)	−78.8 (13.1)	−22.4 (312.6)	−1.8 (28.9)	−74.2 (30.6)**
LDL_3+4_-C							
Baseline	49.5 (18.1)	49.2 (19.6)	45.9 (18.0)	53.2 (18.0)	48.8 (26.1)	56.4 (19.9)	46.3 (22.7)
Post-treatment^b^	52.4 (15.8)	14.9 (8.1)	39.7 (14.1)	18.0 (7.8)	19.2 (13.4)	51.1 (16.1)	22.3 (14.5)
% change from baseline	15.0 (41.4)	−68.5 (18.0)**	−0.9 (57.9)	−64.1 (18.8)**	−55.2 (30.8)**	−4.5 (27.3)	−48.8 (31.2)**

Values are mean (SD). Units are mg/dL. *p < 0.05; **p < 0.0001. ^a^Patients in study 566 were randomised to one of three arms and received either (1) placebo with increase in ATV dose from 10 mg to 80 mg at start of randomised treatment period, (2) alirocumab plus ATV 10 mg, or (3) alirocumab with increase in ATV dose from 10 mg to 80 mg at start of randomised treatment period. ^b^Measurements occurred on Week 12 in Study 565, Week 8 in study 566 and Week 6 in study 1003. *Q2W* every 2 weeks, *ATV* atorvastatin, *IDL* intermediate-density lipoprotein, *LDL-C* low density lipoprotein cholesterol, *LDLr* “LDL real” [i.e. total LDL fraction minus Lp(a) and IDL], *Lp(a)* lipoprotein (a), *SD*, standard deviation

### ApoB/apoA1 ratio

Levels of apoB were significantly reduced in each of the parent studies (Additional file 1: Table S1). Changes in the apoB/apoA1 ratio for these studies have not been reported previously. The apoB/apoA1 ratio was reduced by 50.3 % in study 565, 44.5–47.4 % in study 566 and 46.3 % in study 1003 (*p* < 0.0001 vs. placebo; Additional file 1: Table S2).

### VLDL-C and RLP-C

Alirocumab treatment reduced levels of VLDL-C by 26.1–32.4 % and RLP-C (VLDL_3_-C and IDL-C) by 42.1–52.5 % across the studies (Table 3). Levels of VLDL subfractions VLDL_1+2_-C and VLDL_3_-C were reduced by similar amounts. Triglyceride levels were significantly reduced with alirocumab in studies 565 and 566 (Table 3).

**Table 3:** Changes from baseline in VLDL and remnant-related lipoprotein subfractions and triglycerides

	Study 565		Study 566^a^			Study 1003	
	Placebo	Alirocumab 150 mg Q2W	Placebo + ATV 80 mg	Alirocumab 150 mg Q2W + ATV 10 mg	Alirocumab 150 mg Q2W + ATV 80 mg	Placebo	Alirocumab 150 mg Q2W
	*n =* 31	*n* = 29	*n* = 26	*n* = 26	*n* = 29	*n* = 14	*n* = 16
Total VLDL-C							
Baseline	25.0 (20.5 to 31.5)	24.0 (18.0 to 30.0)	24.0 (20.0 to 29.0)	24.5 (21.0 to 31.0)	21.0 (17.0 to 24.0)	24.0 (18.0 to 33.0)	26.0 (18.5 to 31.5)
Post-treatment^b^	24.0 (19.5 to 32.0)	17.0 (14.0 to 19.0)	20.0 (17.0 to 26.0)	20.0 (17.0 to 23.0)	16.0 (14.0 to 19.0)	27.0 (19.0 to 30.0)	16.5 (15.0 to 20.0)
% change from baseline	5.6 (−19.4 to 19.4)	−32.4 (−37.1 to −17.4)**	−13.5 (−26.5 to 6.7)	−24.1 (−36.4 to 5.3)	−26.1 (−38.7 to −10.0)*	−3.6 (−12.8 to 21.4)	−32.2 (−46.5 to −19.6)*
VLDL_1+2_-C							
Baseline	10.2 (7.8 to 12.2)	9.3 (6.9 to 13.8)	9.9 (7.9 to 13.3)	10.4 (8.3 to 13.9)	7.5 (6.8 to 10.7)	9.1 (6.3 to 14.4)	10.5 (7.5 to 12.5)
Post-treatment^b^	10.2 (7.9 to 14.0)	6.2 (5.1 to 7.7)	7.7 (6.5 to 11.7)	8.8 (6.7 to 10.1)	6.3 (5.3 to 8.5)	9.8 (7.7 to 11.8)	6.4 (5.4 to 8.7)
% change from baseline	12.1 (−21.9 to 27.9)	−34.6 (−41.7 to −20.4)**	−15.1 (−31.1 to 14.7)	−18.9 (−44.6 to 10.6)	−25.7 (−37.8 to −12.1)*	−8.0 (−16.7 to 24.4)	−33.9 (−49.5 to -–3.7)*
VLDL_3_-C							
Baseline	15.0 (12.5 to 18.5)	14.0 (11.0 to 17.0)	14.0 (12.0 to 17.0)	14.0 (12.0 to 17.0)	12.0 (11.0 to 15.0)	14.5 (12.0 to 18.0)	15.0 (12.0 to 18.5)
Post-treatment^b^	14.0 (12.0 to 18.5)	10.0 (8.0 to 12.0)	13.0 (10.0 to 15.0)	11.0 (10.0 to 13.0)	10.0 (8.0 to 11.0)	17.0 (11.0 to 18.0)	10.0 (9.0 to 12.5)
% change from baseline	0.0 (−15.5 to 15.9)	−27.3 (−35.3 to −20.0)**	−14.3 (−21.4 to 6.7)	−28.7 (−33.3 to −9.1)	−23.1 (−36.4 to −16.7)*	0.2 (−12.5 to 11.1)	−32.3 (−42.4 to −18.3)*
IDL-C							
Baseline	16.0 (13.0 to 20.0)	15.0 (12.0 to 19.0)	16.0 (13.0 to 20.0)	17.5 (12.0 to 20.0)	14.0 (11.0 to 20.0)	21.0 (14.0 to 22.0)	22.5 (17.5 to 26.0)
Post-treatment^b^	15.0 (12.5 to 20.0)	7.0 (4.0 to 9.0)	11.5 (9.0 to 16.0)	8.0 (6.0 to 10.0)	6.0 (5.0 to 8.0)	20.0 (16.0 to 24.0)	8.0 (5.5 to 9.5)
% change from baseline	−2.9 (−16.5 to 12.7)	−53.9 (−63.6 to −50.0)**	−22.5 (−42.1 to 7.1)	−51.5 (−63.6 to −37.5)*	−57.1 (−72.7 to −41.7)**	−10.0 (−27.3 to–15.8)	−68.6 (−73.1 to −49.2)**
Triglycerides							
Baseline	132.0 (104.0 to 204.5)	148.0 (102.0 to 194.0)	135.0 (102.0 to 154.0)	149.5 (99.0 to 179.0)	124.0 (90.0 to 149.0)	140.5 (100.0 to 211.0)	131.5 (109.5 to 179.0)
Post-treatment^b^	143.0 (115.0 to 210.5)	94.0 (78.0 to 152.0)	119.0 (87.0 to 180.0)	117.0 (86.0 to 162.0)	92.0 (72.0 to 116.0)	140.0 (93.0 to 162.0)	114.5 (99.0 to 144.0)
% change from baseline	2.4 (−15.9 to 33.9)	−21.7 (−36.8 to 3.4)*	−11.6 (−29.8 to 22.5)	−13.5 (−31.3 to 18.6)	−24.6 (−39.0 to −8.4)*	−7.0 (−26.3 to 6.6)	−22.2 (−31.3 to −1.9)
RLP-C^c^							
Baseline	30.0 (26.0 to 37.0)	29.0 (24.0 to 35.0)	32.5 (24.0 to 40.0)	33.0 (24.0 to 36.0)	25.0 (21.0 to 35.0)	36.0 (28.0 to 43.0)	37.5 (30.5 to 44.5)
Post-treatment^b^	29.0 (25.0 to 38.0)	18.0 (13.0 to 20.0)	24.0 (18.0 to 32.0)	19.0 (17.0 to 22.0)	16.0 (14.0 to 18.0)	36.0 (32.0 to 39.0)	18.5 (15.0 to 22.0)
% change from baseline	−4.4 (−14.5 to 9.3)	−42.1 (−50.0 to −34.5)**	−17.7 (−31.4 to 9.5)	−38.7 (−50.0 to −26.7)*	−42.1 (−55.0 to −29.4)**	−8.5 (−18.8 to 18.2)	−52.5 (−61.3 to −34.1)**

Values are median (Q1:Q3), mg/dL. *p < 0.05; **p < 0.0001. ^a,b^See footnotes to Table 2. ^c^RLP-C consists of VLDL_3_-C + IDL-C. *Q2W* every 2 weeks, *ATV* atorvastatin, *IDL-C* intermediate-density lipoprotein cholesterol, *RLP-C* remnant lipoprotein cholesterol, *VLDL-C* very low-density lipoprotein cholesterol

### Apo CII and CIII

To investigate further the observed reductions in VLDL-C, the effect of alirocumab on serum levels of apoCII and CIII was assessed. Reductions from baseline of 9.4–27.8 % in apoCII and 14.5–19.1 % apoCIII were observed with alirocumab treatment (Table 4). There was no difference in the ratios of apoCII/VLDL-C or apoCIII/VLDL-C at baseline vs. post-treatment (Additional file 1: Table S3).

**Table 4:** Changes from baseline in apoCII and apoCIII

	Study 565		Study 566^a^			Study 1003	
	Placebo	Alirocumab 150 mg Q2W	Placebo + ATV 80 mg	Alirocumab 150 mg Q2W + ATV 10 mg	Alirocumab 150 mg Q2W + ATV 80 mg	Placebo	Alirocumab 150 mg Q2W
	*n* = 30	*n* = 28	*n* = 27	*n* = 26	*n* = 29	*n* = 14	*n* = 16
ApoCII							
Baseline	4.7 (2.1)	5.0 (2.1)	5.0 (1.8)	4.9 (2.1)	5.3 (2.0)	4.8 (2.3)	4.4 (1.9)
Post- treatment^b^	5.3 (2.8)	3.8 (1.4)	4.3 (1.8)	4.2 (1.4)	3.7 (1.8)	4.7 (2.0)	3.7 (1.0)
% change from baseline	12.7 (32.6)	−18.2 (28.3)**	−7.6 (36.3)	−8.8 (23.0)	−27.8 (20.6)*	1.6 (15.5)	−9.4 (28.1)
ApoCIII							
Baseline	11.0 (4.0)	10.5 (3.2)	11.0 (4.6)	11.2 (4.1)	11.5 (4.5)	12.3 (4.5)	11.5 (4.7)
Post- treatment^b^	12.2 (6.1)	8.6 (2.5)	10.0 (3.5)	9.8 (2.7)	8.9 (3.1)	11.6 (3.2)	9.4 (2.3)
% change from baseline	12.1 (37.5)	−16.1 (20.1)**	−2.0 (34.1)	−9.4 (19.5)	−19.1 (17.7)*	−1.8 (19.3)	−14.5 (19.7)

Values are mean (SD), units are mg/dL. *p < 0.05; **p < 0.0001.^a,b^See footnotes to Table 2. *Q2W* every 2 weeks, *apo* apolipoprotein, *ATV* atorvastatin, *SD* standard deviation

### Pooled analysis

A pooled analysis combining data from the three trials was generally consistent with the individual study results (Additional file 1: Table S4).

## Discussion

Alirocumab significantly reduced cholesterol levels across the spectrum of LDL subfractions LDL_1_-C, LDL_2_-C, LDL_3_-C and the pool of LDL_3+4_-C (sum of smaller, denser LDL subfractions). Notably, reductions in LDL subfractions were overall consistent between patients with heFH and primary hyperlipidemia. Reductions in LDL-C subfractions in the placebo arm of study 566 were larger than the other two studies, most likely due to the concomitant atorvastatin dose increase from 10 mg at baseline to 80 mg at randomisation. Small differences between the studies in terms of the percentage change of lipoprotein parameters following alirocumab treatment may be explained by differences in the patient populations, e.g. baseline lipids, HeFH/non-FH, sex distribution and background therapies [14–16].

With regards to effects on the densest LDL subfraction (LDL_4_-C), significant absolute reductions were observed in all alirocumab groups vs. placebo. Mean percentage reductions in this parameter were inconsistent between studies; most likely this is due to low baseline levels and small absolute changes resulting in high levels of variation. In a randomised study, doubling the atorvastatin dose or adding ezetimibe was reported to reduce dense LDL particles by a lesser extent than less dense particles [20]. One explanation for this is that the densest particles have a lower affinity for the LDL receptor [20]; however, it remains to be established why LDL_4_ has the lowest levels of clearance.

The significant reduction from baseline in the ratio of apoB/apoA1 ratio (range 45–50 % across the studies) suggests further improvement in the atherogenic cholesterol profile with alirocumab treatment. Multiple studies have demonstrated that the apoB/apoA1 ratio is a more sensitive predictor of future cardiovascular events than individual lipoproteins or ratios of cholesterol values [21–23]. Levels of apoB may be a more accurate predictor of cardiovascular risk than LDL-C, as apoB more closely estimates the number of circulating LDL particles [24]. A low level of apoA1 is also a cardiovascular risk factor and reflects low serum levels of HDL particles [23].

Alirocumab reduced lipoprotein cholesterol across multiple atherogenic lipoprotein fractions including VLDL-C and IDL-C. This finding is consistent with the fact that all are apoB-containing lipoproteins, some of which may be cleared by LDL receptors, as is LDL-C. It cannot be discerned from these data whether or not alirocumab potentiates RLP clearance by increasing expression of the LDL receptor-related protein or heparin sulfate proteoglycans, both of which are involved in the binding and clearance of remnants [25–27]. RLPs are products of VLDL lipolysis and include VLDL_3_, the most dense VLDL subclass, and IDL, the direct precursor of LDL [28]. There is accumulating evidence demonstrating a causal association between elevated RLP-C levels and an increased risk of ischaemic heart disease [28, 29]. Reductions in RLP-C with alirocumab (24–44 %, placebo-corrected) appears similar if not greater than reductions observed with statins (25 %) [30]. The reductions in apoCII and apoCIII observed in the three studies may be a manifestation of either increased clearance or reduced production/secretion of VLDL particles. The observation that apoCII/VLDL-C and apoCIII/VLDL-C ratios do not change significantly on therapy vs. baseline suggests alirocumab does not impact the synthesis of apoCII or apoCIII.

Limitations of the current analysis include its *post hoc* nature; the findings should be regarded as hypothesis-generating. In addition, overall patient numbers were relatively small and patients were treated for a limited duration. Although the study populations represent typical patients being treated for heFH or non-FH, there were few patients with diabetes (11 % of the randomised populations in the three studies), hence it is not possible to confirm the effects of alirocumab on lipoprotein fractions in such patients. Analysis of effects of lipid-modifying therapies on LDL-C particle composition may aid understanding of treatment mode-of-action as well as further understanding of the derived clinical benefit of the treatment. However, the clinical utility of measuring lipoprotein subfractions in risk assessment remains to be elucidated, and current guidelines do not advocate such an approach because of a lack of supportive clinical trial data [6].

## Conclusions

To conclude, in this *post hoc* analysis of three Phase II trials, alirocumab 150 mg Q2W reduced cholesterol across the spectrum of atherogenic lipoproteins separated by VAP (including LDL, IDL and VLDL subfractions). Reductions were consistent in both patients with heFH and primary hyperlipidaemia. The potential clinical impact of alirocumab on these lipid variables warrants further investigation. Alirocumab is being assessed in the Phase III ODYSSEY clinical trial program (https://clinicaltrials.gov/).

## Methods

Patient samples from three Phase II multicentre, double-blind, parallel-group, placebo-controlled trials were used in this analysis: study 565 (NCT01288443) [14] and study 566 (NCT01288469) [15] in patients with non-familial hypercholesterolaemia (non-FH) and study 1003 (NCT01266876) [16] in patients with heterozygous familial hypercholesterolaemia (heFH). Studies 565 and 1003 had a 12-week double blind period; study 566 had an 8-week double blind period. All were multicenter trials conducted in the US (study 1003 was also conducted in Canada). The study protocols were approved by the institutional review boards at each study center and appropriate ethical approval was obtained. Written informed consent was obtained from all participants in the studies. Patients with LDL-C ≥2.59 mmol/L (100 mg/dL) at baseline on statins (± ezetimibe in study 1003) were treated with subcutaneous alirocumab 50–150 mg every 2 weeks (Q2W) or 150–300 mg every 4 weeks, depending on the study. The 150 mg Q2W alirocumab dose was common to all three trials and is the focus of this analysis.

Background statin was stable in studies 565 and 1003. Study 566 comprised three treatment arms: alirocumab 150 mg Q2W plus atorvastatin 10 mg, alirocumab 150 mg Q2W with atorvastatin dose increase from 10 to 80 mg at randomisation, and placebo with the same atorvastatin dose increase.

### Measurement methodology

Cholesterol content of major lipoproteins and subfractions was analysed using VAP (Atherotech Diagnostics Laboratory, Birmingham, AL, USA). VAP is a validated technique that separates lipoproteins based on density by single vertical-spin density gradient ultracentrifugation [10, 11]. The amount of cholesterol of each lipoprotein and subfraction is quantified using a continuous flow analyser and a cholesterol-specific enzymatic/spectrophotometric method. The accuracy and reproducibility of the VAP method are within the requirements of the US Centers for Disease Control-National Heart, Lung, and Blood Institute Lipid Standardization Program [10, 11] and comply with the standards established by the National Cholesterol Education Program guidelines [9]. Accuracy of the VAP method is monitored on an on-going basis by split-sample comparisons with results obtained using beta quantification at the Core Laboratories for Clinical Studies at Washington University, St. Louis, MO [9, 31].

Total LDL-C was determined as the directly measured equivalent of the original definition by Friedewald [32] representing non-HDL-C minus VLDL-C, the sum of cholesterol carried in biologic or “real” LDL (LDLr-C), and IDL (IDL-C). LDLr-C was further separated into four subfractions using VAP: LDL_1_-C, LDL_2_-C, LDL_3_-C, and LDL_4_-C (increasing in density from subclass 1 through 4). The two densest subfractions, LDL_3_-C and LDL_4_-C, were analysed individually and also pooled (LDL_3+4_-C), representing the sum of dense LDL subfraction cholesterol since LDL_4_-C typically exists at substantially lower concentrations than the other three LDL subfractions. LDL_1_-C and LDL_2_-C were also pooled, representing the sum of the more buoyant, lower density LDL subfraction cholesterol. Other lipoproteins measured using VAP included VLDL-C and its subfractions VLDL_1_-C, VLDL_2_-C, and VLDL_3_-C (again, increasing in density from subclass 1 through 3), IDL-C and its subfractions IDL_1−_C and IDL_2−_C (with subclass 2 being more dense), and total RLP cholesterol (RLP-C, comprising VLDL_3_-C + IDL-C).

The ratio of apoB to apoAI levels was calculated based on measurements determined in the respective parent studies using conventional techniques, as were triglyceride levels [14–16]. ApoCII and apoCIII concentrations were measured at Atherotech Diagnostics Laboratory (Birmingham, AL, USA) using reagent kits obtained from Randox Laboratories Limited, UK (apoCII, Cat. No. LP3866; apoCIII, Cat. No. LP3865) and an Architect ci8200 analyser (Abbott Laboratories). The immunoassay methods are based on the reaction of a sample containing human apoCII (or CIII) and specific antiserum to apoCII (or CIII) to form an insoluble complex, the concentration of which can be measured turbidimetrically at 340 nm. Both assays were validated for analytical performance.

### Statistical analyses

All statistical analyses were performed using R version 3.0.2. Mean levels of cholesterol in lipoprotein fractions and subfractions, triglycerides, apoCII and apoCIII, and the apoB/apoA1 ratio were assessed in patients receiving alirocumab 150 mg Q2W vs. placebo at baseline and at Week 12 for study 565, Week 8 for study 566 and Week 6 for study 1003. Study 1003 had a Week 12 endpoint; however, 6-week data were used for this analysis due to reduced numbers of patients with available samples at 12 weeks (*n* = 17; all dosing groups) compared with 6 weeks (*n* = 75; all dosing groups). To determine if percentage changes from baseline to the 6–12 week time points were significant for alirocumab vs. placebo, analysis of covariance was performed with the baseline value as a covariate. Significance is considered at alpha ≤0.05. P-values were not adjusted for multiplicity and are presented for descriptive purposes only.

## Additional file

Additional file 1: Table S1. Changes from baseline in lipids and lipoproteins as measured using conventional methods in the parent studies. **Table S2.** Change from baseline in apoB/apoAI ratio. **Table S3.** Changes from baseline in ratios of apoCII/VLDL-C and apoCIII/VLDL-C. **Table S4.** Pooled data from across the three studies (565, 566, 1003) for changes from baseline in cholesterol content of lipoprotein subfractions, apoB/apoA1 ratio, and levels of apo CII and CIII. (DOCX 45 kb)

## Abbreviations

Apo: apolipoprotein
ATV: atorvastatin
C: cholesterol
FH: familial hypercholesterolaemia
HDL: high-density lipoprotein
IDL: intermediate-density lipoprotein
LDL: low-density lipoprotein
LDLr: “real” LDL
PCSK9: Proprotein convertase subtilisin/kexin type 9
Q2W: every 2 weeks
RLP: remnant lipoprotein
SD: standard deviation
VAP: vertical auto profile
VLDL: very low-density lipoprotein
