# Supplementary material for: Effect of alirocumab on specific lipoprotein non-high-density lipoprotein cholesterol and subfractions as measured by the vertical auto profile method: analysis of 3 randomized trials versus placebo
Source: Lipids Health Dis. 2016 Feb 13;15:28. doi: 10.1186/s12944-016-0197-4 (PMC4752766; doi:10.1186/s12944-016-0197-4)
Supplement: Additional file 1: Table S1. — Changes from baseline in lipids and lipoproteins as measured using conventional methods in the parent studies. Table S2. Change from baseline in apoB/apoAI ratio. Table S3. Changes from baseline in ratios of apoCII/VLDL-C and apoCIII/VLDL-C. Table S4. Pooled data from across the three studies (565, 566, 1003) for changes from baseline in cholesterol content of lipoprotein subfractions, apoB/apoA1 ratio, and levels of apo CII and CIII. (DOCX 45 kb) [file 12944_2016_197_MOESM1_ESM.docx]

# Supplementary appendix

Table S1 Changes from baseline in lipids and lipoproteins as measured using conventional methods in the parent studies

|  | **Study 565^†^** | | **Study 566^‡^** | | | **Study 1003^§^** | |
| --- | --- | --- | --- | --- | --- | --- | --- |
|  | **Placebo**  **n = 31** | **Alirocumab 150 mg Q2W**  **n = 29** | **Placebo + ATV 80 mg**  **n = 29** | **Alirocumab 150 mg Q2W**  **+ ATV 10 mg**  **n = 29** | **Alirocumab 150 mg Q2W + ATV 80 mg**  **n = 30** | **Placebo + ATV 80 mg**  **n = 15** | **Alirocumab  150 mg Q2W**  **n = 16** |
| LDL-C | –5.1 (3.1) | –72.4 (3.2)** | –17.3 (3.5) | −66.2 (3.5)** | –73.2 (3.5)** | –10.7 (5.0) | –67.9 (4.9)** |
| Non-HDL-C | –2.2 (2.9) | –62.5 (3.0)** | −22.3 (−31.4 to −3.7) | −58.3 (−63.9 to −50.2)** | −63.9 (−73.9 to −56.1)** | –11.3 (4.7) | –57.9 (4.6)** |
| ApoB | 2.2 (2.9) | –56.1 (2.9)** | −12.0 (−23.6 to −3.5) | −54.4 (−60.2 to −48.3)** | −58.0 (−67.1 to −46.1)** | –6.4 (4.2) | –50.2 (4.0)** |
| TGs | 9.7 (–15.0 to 30.7) | –18.9 (–31.7 to  –6.1)* | −11.9 (−30.4 to 14.3) | −4.0 (−30.5 to 17.4) | −24.7 (−40.3 to −4.4)* | –10.6 (–28.5 to 9.5) | –16.2 (–30.7 to 25.1) |
| HDL-C | –1.0 (2.3) | 5.5 (2.4) | −3.6 (2.3) | 2.6 (2.3) | 5.8 (2.3)* | 2.2% (3.7) | 12.3 (3.6)* |
| ApoA1 | 0.0 (–7.2 to 5.3) | 1.4 (–2.1 to 5.4) | −5.2 (2.3) | 0.4 (2.3) | −2.2 (2.3) | –5.3% (3.1) | 8.8 (2.9)* |

Values are mean (SE) or median (Q1:Q3) % changes from baseline to Week 12 in studies 565 and 1003, and from baseline to Week 8 in study 566. Patients in study 566 were randomised to one of three arms and received either (1) placebo with increase in ATV dose from 10 mg to 80 mg at start of randomised treatment period, (2) alirocumab plus ATV 10 mg or (3) alirocumab with increase in ATV dose from 10 mg to 80 mg at start of randomised treatment period.

*p < 0.05; ** p < 0.0001 vs. placebo. ^†^McKenney et al., 2012; ^‡^Roth et al., 2012; ^ǁ^Stein et al., 2012. Refer to reference list in main manuscript. *Q2W*, every 2 weeks; *apo*, apolipoprotein; *ATV,* atorvastatin; *HDL-C,* high-density lipoprotein cholesterol; *LDL-C*, low-density lipoprotein cholesterol; *SE,* standard error; *TGs,* triglycerides.

Table S2 Change from baseline in apoB/apoAI ratio

|  | **Study 565** | | **Study 566^†^** | | | **Study 1003** | |
| --- | --- | --- | --- | --- | --- | --- | --- |
|  | **Placebo**  **n = 31** | **Alirocumab 150 mg Q2W**  **n = 29** | **Placebo + ATV 80 mg**  **n = 26** | **Alirocumab 150 mg Q2W**  **+ ATV 10 mg**  **n = 26** | **Alirocumab 150 mg Q2W + ATV 80 mg**  **n = 29** | **Placebo**  **n = 14** | **Alirocumab  150 mg Q2W**  **n = 16** |
| Baseline | 0.7 (0.1) | 0.7 (0.2) | 0.7 (0.1) | 0.7 (0.1) | 0.6 (0.1) | 0.8 (0.1) | 0.8 (0.1) |
| Post-treatment^‡^ | 0.7 (0.2) | 0.3 (0.1) | 0.6 (0.2) | 0.4 (0.1) | 0.3 (0.1) | 0.7 (0.1) | 0.4 (0.2) |
| % change from baseline | –1.7 (12.6) | –50.3 (11.7)** | –7.2 (23.4) | –44.5 (13.4)** | –47.4 (14.5)** | –5.8 (11.7) | –46.3 (20.3)** |

Values are mean (SD).

**p < 0.0001 vs. placebo.^†^Patients in study 566 were randomised to one of three arms and received either (1) placebo with increase in ATV dose from 10 mg to 80 mg at start of randomised treatment period, (2) alirocumab plus ATV 10 mg, or (3) alirocumab with increase in ATV dose from 10 mg to 80 mg at start of randomised treatment period. ^‡^Study 565, Week 12; study 566, Week 8; study 1003, Week 6. *Q2W*, every 2 weeks; Apo, apolipoprotein; ATV, atorvastatin; *SD*, standard deviation.

Table S3 Changes from baseline in ratios of apoCII/VLDL-C and apoCIII/VLDL-C

|  | **Study 565** | | **Study 566^†^** | | | **Study 1003** | |
| --- | --- | --- | --- | --- | --- | --- | --- |
|  | **Placebo**  **n = 30** | **Alirocumab**  **150 mg Q2W**  **n = 28** | **Placebo  + ATV 80 mg**  **n = 27** | **Alirocumab  150 mg Q2W**  **+ ATV 10 mg**  **n = 26** | **Alirocumab  150 mg Q2W  + ATV 80 mg**  **n = 29** | **Placebo**  **n = 14** | **Alirocumab  150 mg Q2W**  **n = 16** |
| ApoCII/VLDL-C |  |  |  |  |  |  |  |
| Baseline | 0.18 (0.06) | 0.21 (0.05) | 0.2 (0.1) | 0.19 (0.07) | 0.24 (0.09) | 0.18 (0.06) | 0.17 (0.04) |
| Post-treatment‡ | 0.2 (0.07) | 0.23 (0.07) | 0.19 (0.05) | 0.21 (0.06) | 0.22 (0.08) | 0.19 (0.07) | 0.21 (0.03) |
| % change from baseline | 11.9 (24.4) | 13.8 (26.2) | 0.3 (22.8) | 14.3 (20.6)* | –4.0 (23.8) | 4.3 (21.3) | 26.3 (23.9)* |
| ApoCIII/VLDL-C |  |  |  |  |  |  |  |
| Baseline | 0.42 (0.12) | 0.45 (0.11) | 0.43 (0.11) | 0.45 (0.14) | 0.52 (0.22) | 0.48 (0.09) | 0.45 (0.12) |
| Post-treatment^‡^ | 0.46 (0.1) | 0.52 (0.13) | 0.46 (0.12) | 0.5 (0.12) | 0.54 (0.16) | 0.48 (0.12) | 0.45 (0.12) |
| % change from baseline | 10.3 (21.0) | 19.0 (22.9) | 7.8 (26.6) | 14.6 (20.1) | 9.1 (22.6) | 0.6 (24.4) | 22.2 (27.7)* |

Values are mean (SD).

*p < 0.05 vs. placebo. ^†^Patients in study 566 were randomised to one of three arms and received either (1) placebo with increase in ATV dose from 10 mg to 80 mg at start of randomised treatment period, (2) alirocumab plus ATV 10 mg or (3) alirocumab with increase in ATV dose from 10 mg to 80 mg at start of randomised treatment period. ^‡^Study 565, Week 12; study 566, Week 8; study 1003, Week 6. *Q2W*, every 2 weeks; *Apo*, apolipoprotein *ATV*, atorvastatin; *SD*, standard deviation; *VLDL-C,* very low-density lipoprotein cholesterol

Table S4 Pooled data from across the three studies (565, 566, 1003) for changes from baseline in cholesterol content of lipoprotein subfractions, apoB/apoA1 ratio, and levels of apo CII and CIII

| Lipoprotein subfractions, mg/dL | Pooled data | |
| --- | --- | --- |
|  | Placebo n = 72^a^ | Alirocumab 150 mg Q2W n = 100^†^ |
| LDL total |  |  |
| Baseline | 121.8 (29.1) | 120.9 (28.8) |
| Post-treatment^‡^ | 110.3 (33.1) | 42.8 (22.9) |
| Mean (SD) change from baseline, % | –7.5 (25.1) | -64.1 (18.4) |
| P-value |  | <0.0001 |
| LDL-R |  |  |
| Baseline | 95.7 (25.6) | 94.4 (25.1) |
| Post-treatment^‡^ | 86.1 (27.7) | 27.9 (19.7) |
| Mean (SD) change from baseline, % | –7.2 (28.4) | –70.6 (19.4) |
| P-value |  | <0.0001 |
| LDL_1_-C |  |  |
| Baseline | 19.8 (7.6) | 19.5 (8.5) |
| Post-treatment^‡^ | 17.1 (8.8) | 4.9 (4.3) |
| Mean (SD) change from baseline, % | –7.4 (47.6) | –64.0 (110.0) |
| P-value |  | 0.0001 |
| LDL_2_-C |  |  |
| Baseline | 26.2 (14.3) | 25.3 (14.2) |
| Post-treatment^‡^ | 20.8 (14.5) | 4.9 (7.5) |
| Mean (SD) change from baseline, % | –8.3 (98.9) | –82.8 (21.4) |
| P-value |  | <0.0001 |
| LDL_3_-C |  |  |
| Baseline | 39.4 (14.6) | 37.9 (14.3) |
| Post-treatment^‡^ | 35.9 (13.0) | 11.3 (9.0) |
| Mean (SD) change from baseline, % | –0.2 (45.0) | –68.4 (25.7) |
| P-value |  | <0.0001 |
| LDL_4_-C |  |  |
| Baseline | 10.4 (7.9) | 11.7 (10.0) |
| Post-treatment^‡^ | 12.3 (8.6) | 6.8 (3.3) |
| Mean (SD) change from baseline, % | 78.9 (182.9) | 13.7 (213.3) |
| P-value |  | 0.1596 |
| LDL_1+2_-C |  |  |
| Baseline | 46.0 (19.7) | 44.8 (20.2) |
| Post-treatment^‡^ | 37.9 (21.4) | 9.8 (11.0) |
| Mean (SD) change from baseline, % | –11.1 (46.4) | –62.3 (169.1) |
| P-value |  | 0.0201 |
| LDL_3+4_-C |  |  |
| Baseline | 49.7 (18.5) | 49.7 (21.6) |
| Post-treatment^‡^ | 48.2 (17.1) | 18.1 (11.1) |
| Mean (SD) change from baseline, % | 6.0 (46.3) | –60.3 (25.5) |
| P-value |  | <0.0001 |
| ApoB/A1 |  |  |
| Baseline | 0.7 (0.1) | 0.7 (0.2) |
| Post-treatment^‡^ | 0.7 (0.2) | 0.4 (0.1) |
| Mean (SD) change from baseline, % | –4.5 (17.2) | –47.3 (14.5) |
| P-value |  | <0.0001 |
| VLDL-C |  |  |
| Baseline | 24.5 (20.0 to 32.5) | 23.0 (18.0 to 30.5) |
| Post-treatment^‡^ | 23.5 (18.0 to 29.5) | 17.0 (14.0 to 20.0) |
| Mean (SD) change from baseline, % | –3.9 (–22.5 to 19.4) | –27.1 (–38.9 to –15.8) |
| P-value |  | <0.0001 |
| VLDL_1+2_-C |  |  |
| Baseline | 9.9 (7.7 to 13.2) | 9.5 (7 to 12.7) |
| Post-treatment^‡^ | 9.4 (7.2 to 12.1) | 7.1 (5.5 to 8.8) |
| Mean (SD) change from baseline, % | –1.6 (–28.0 to 24.4) | –27.8 (–42.9 to –12.2) |
| P-value |  | <0.0001 |
| VLDL_3_-C |  |  |
| Baseline | 14.5 (12.0 to 18.0) | 14.0 (11.0 to 17.0) |
| Post-treatment^‡^ | 13.5 (11.0 to 18.0) | 10.0 (9.0 to 12.0) |
| Mean (SD) change from baseline, % | –5.1 (–18.8 to 10.8) | –25.8 (–35.7 to –15.4) |
| P-value |  | <0.0001 |
| IDL-C |  |  |
| Baseline | 16.5 (13.0 to 21.0) | 17.0 (12.0 to 21.0) |
| Post-treatment^‡^ | 15.0 (10.5 to 20.5) | 7.0 (5.0 to 9.0) |
| Mean (SD) change from baseline, % | –10.6 (–28.6 to 13.9) | –57.1 (–68.6 to –42.3) |
| P-value |  | <0.0001 |

| Triglycerides |  |  |
| --- | --- | --- |
| Baseline | 135.0 (102.5 to 202.0) | 132.0 (97.0 to 179.0) |
| Post-treatment^‡^ | 137.0 (99.5 to 190.0) | 101.0 (82.0 to 151.5) |
| Mean (SD) change from baseline, % | 0.2 (–24.7 to 23.4) | –21.7 (–35.8 to 3.8) |
| P-value |  | 0.0008 |
| RLP-C |  |  |
| Baseline | 32.5 (25.5 to 40.0) | 30.5 (23.0 to 36.0) |
| Post-treatment^‡^ | 28.5 (23.0 to 37.0) | 17.5 (14.0 to 21.0) |
| Mean (SD) change from baseline, % | –7.2 (–21.0 to 11.0) | –42.5 (–52.9 to –31.3) |
| P-value |  | <0.0001 |
| ApoCII |  |  |
| Baseline | 4.8 (2.0) | 4.9 (2.0) |
| Post-treatment^‡^ | 4.8 (2.3) | 3.9 (1.4) |
| Mean (SD) change from baseline, % | 2.8 (32.5) | –17.1 (25.7) |
| P-value |  | <0.0001 |
| ApoCIII |  |  |
| Baseline | 11.3 (4.3) | 11.2 (4.1) |
| Post-treatment^‡^ | 11.3 (4.8) | 9.1 (2.7) |
| Mean (SD) change from baseline, % | 4.0 (33.6) | –15.0 (19.3) |
| P-value |  | <0.0001 |
| ApoCII/VLDL-C |  |  |
| Baseline | 0.2 (0.1) | 0.2 (0.1) |
| Post-treatment^‡^ | 0.2 (0.1) | 0.2 (0.1) |
| Mean (SD) change from baseline, % | 6.0 (23.5) | 10.7 (25.6) |
| P-value |  | 0.0866 |
| ApoCIII/VLDL-C |  |  |
| Baseline | 0.4 (0.1) | 0.5 (0.2) |
| Post-treatment^‡^ | 0.5 (0.1) | 0.5 (0.1) |
| Mean (SD) change from baseline, % | 7.4 (23.8) | 15.5 (23.1) |
| P-value |  | 0.0072 |

Mean (SD) are reported for continuous normally distributed variables, while median (interquartile range) are reported for non-normally distributed variables. Units are mg/dL.

^†^Pooled data for pool of studies 565, 566 and 1003. Patients included from studies 565 and 1003 all received either placebo or alirocumab 150 mg Q2W. Patients in study 566 were randomised to one of three arms and received either (1) placebo with increase in ATV dose from 10 mg to 80 mg at start of randomised treatment period, (2) alirocumab 150 mg Q2W plus ATV 10 mg or (3) alirocumab 150 mg Q2W with increase in ATV dose from 10 mg to 80 mg at start of randomised treatment period. ^‡^Study 565, Week 12; study 566, Week 8; study 1003, Week 6. *Q2W,* every 2 weeks; *Apo,* apolipoprotein; *IDL-C*, intermediate-density lipoprotein cholesterol *LDL-C*, low-density lipoprotein cholesterol; *LDLr,* “LDL real” [i.e. total LDL fraction minus Lp(a) and intermediate density lipoprotein]; *Lp(a),* lipoprotein (a); *RLP-C,* remnant-like particle cholesterol.*SD*, standard deviation; *VLDL-C*, very low-density lipoprotein cholesterol.
